# Mobility of the syntaxin PEN1 in *Arabidopsis* reflects functional specialization of the conserved SYP12 clade

**DOI:** 10.1080/15592324.2022.2084278

**Published:** 2022-06-12

**Authors:** Mengqi Liu, Hector M. Rubiato, Mads E Nielsen

**Affiliations:** a University of Copenhagen, Faculty of Science, CPSC, Department of Plant and Environmental Sciences, Frederiksberg C Denmark; b Universidad Politécnica de Madrid, Campus de Montegancedo, Centre for Plant Biotechnology and Genomics (UPM-INIA), Madrid, Spain

**Keywords:** syntaxin, membrane trafficking, powdery mildew, innate immunity

## Abstract

Plant innate immunity toward cell-wall penetrating filamentous pathogens relies on the conserved SYP12 clade of secretory syntaxins. In *Arabidopsis*, the two closely related SYP12 clade members, PEN1 and SYP122, play an overlapping role in this general immunity, which can be complemented by two SYP12 clade members from *Marchantia* (MpSYP12A and MpSYP12B). However, in addition to the conserved SYP12 clade function, PEN1 alone mediates pre-invasive immunity toward powdery mildew fungi, which likely reflects a specialization of its functionality. Here, we show that the PEN1-specific specialization in immunity correlates with a continuous BFA-sensitive recycling and the ability to accumulate strongly at the growing cell plate. This contrasts with the behavior of SYP122, MpSYP12A, and MpSYP12B, all being more stable at the plasma membrane. We suggest that the highly mobile SYP12 specialization observed for PEN1 is required for a fast pre-invasive immune response to resist attack from powdery mildew fungi.

## Introduction

To resist attack by filamentous pathogens, plants respond by forming cell wall fortifications that hinder the pathogen from entering the host cell.^1^ These defense structures appear to be ancient defense mechanisms that provide general immunity to filamentous pathogens in land plants.^2–5^ Recently, we have shown that in *Arabidopsis*, the two closely related SYP12 clade members of plant secretory syntaxins, PEN1 (SYP121) and SYP122, are indispensable for the formation of these conserved defense structures. This general immunity function is shared by the SYP12 clade members of the early diverging land plant *Marchantia polymorpha* and thus spans some 470 My of independent evolution.^6^ However, PEN1 has a specific function in pre-invasive immunity against powdery mildew fungi, which correlates with its ability to accumulate at unsuccessful attack sites.^7^ We found that the function of pre-invasive immunity against powdery mildew fungi is not shared by the other SYP12 clade members, suggesting that PEN1 has undergone further specialization in plant immunity.

## Materials and Methods

### Plant growth

Plants of *Arabidopsis thaliana* were grown at 21°C, with 8 h of light at 125 micro-Einstein s^−1^m^−2^ per day. All plant lines were previously reported.^6^

### Fluorescent protein and callose detection using confocal microscopy

Samples were examined using a 63× water immersion lens mounted on a Leica CLSM TCS SP5 confocal microscope. Treatments with BFA (50 µM) and FM4-64 (1 µM) were for 1 h at room temperature. For the detection and localization of the fluorophores and stains, GFP and FM4-64 were excited at 488 nm and detected at 500–520 nm and 600–650 nm, respectively, and RFP was excited at 543 nm and detected at 575–595 nm. The cell plates were examined using a 63× water immersion lens mounted on a Leica Stellaris 8 confocal microscope. GFP were excited at 473 nm and detected at HyD X 2 (478 nm – 529 nm), and RFP was excited at 554 nm and detected at HyD X 2 (559 nm – 604 nm). Imaging data were collected at the Center for Advanced Bioimaging (CAB) Denmark, University of Copenhagen. Image overlays and contrast enhancement were performed using image processing software (GIMP 2). Protein alignment was done using CLC Main Workbench 22 software.

## Results

**Figure 1.**
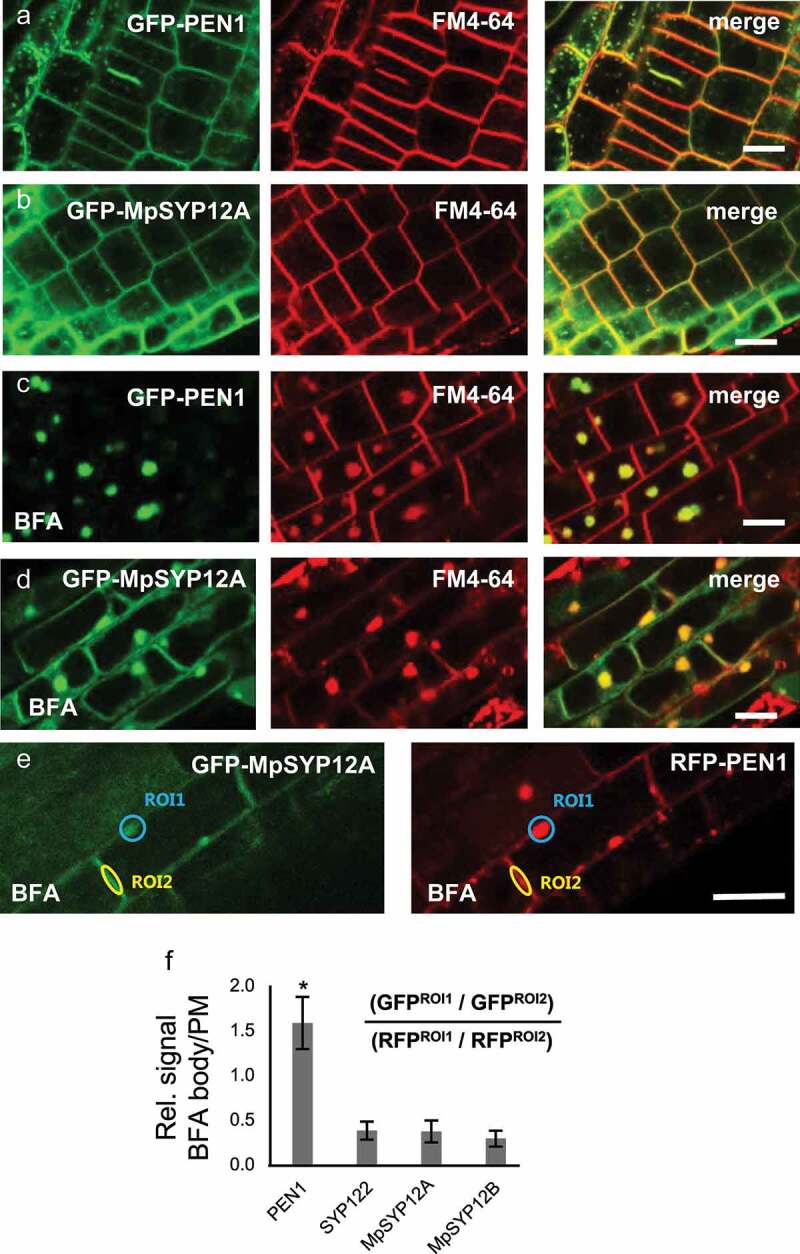
Localization of SYP12 clade members in response to BFA. (a–d) Roots of plants expressing (a and c) GFP-PEN1 or (b and d) GFP-MpSYP12A and stained with FM4-64 (a and b) before and (c and d) after treatment with 50 µM BFA. (e) Roots of plants expressing both GFP-MpSYP12A and RFP-PEN1 after treatment with BFA. Bars = 10 µm. (f) Quantification of SYP12 localization in response to BFA, relative to RFP-PEN1. Signal intensities of BFA body (ROI1) and plasma membrane (PM) (ROI2), were used to calculate the BFA body/PM signal ratios. All values are mean ±SD (n = 18 cells). * indicate significantly different values at P ≤ .001 estimated using student t-test.

To follow up on our studies of the conserved SYP12 clade function in plant innate immunity, we analyzed the localization of the four SYP12 clade members, PEN1 and SYP122 (from *Arabidopsis*), and, MpSYP12A and MpSYP12B (from *Marchantia*), in *Arabidopsis* in response to the fungal toxin brefeldin A (BFA), which blocks the recycling of endocytosed proteins on their way back to the plasma membrane [PM; 8]. Previously, we have shown that the localization of PEN1 is highly sensitive to BFA, whereas the localization of SYP122 is largely unaffected.^9^ In line with what we reported earlier, BFA clearly affected the localization of PEN1, from being predominantly at the PM to being almost exclusively in BFA bodies. In contrast, BFA had only a mild effect on the localization of MpSYP12A and MpSYP12B, which was similar to SYP122 (Figure 1a-d; Supplementary Fig. S1). To better evaluate the effect of BFA on the localization of the SYP12s, we used RFP-PEN1 as an internal control and quantified the relative signal intensity at the PM and BFA bodies for all four SYP12s. The results show that MpSYP12A and MpSYP12B behave like SYP122 in response to BFA and do not mimic the continuous recycling of PEN1 (Figure 1e –f).

**Figure 2.**
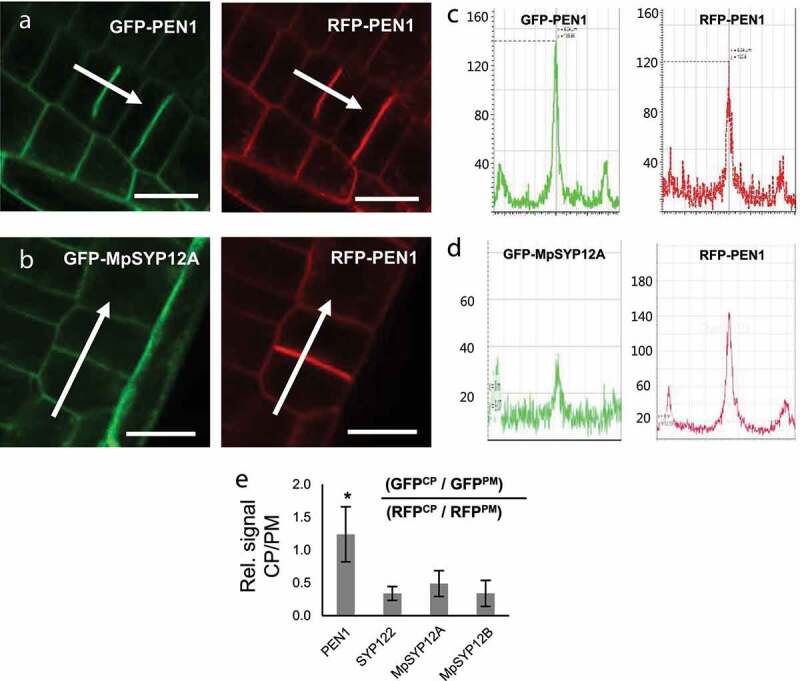
Localization of SYP12 clade members at the forming cell plate. (a and b) Roots of plants expressing (a) GFP-PEN1 or (b) GFP-MpSYP12A in combination with RFP-PEN1 as reference. Bars = 10 µm. (c and d) Signal intensities of GFP and RFP (from a and b, respectively) across the growing cell plate. (e) Calculated signal intensities of SYP12s at the growing cell plate relative to RFP-PEN1. Signal intensities at the growing cell plate (CP) and PM, were used to calculate the CP/PM signal ratios. All values are mean ±SD (n = 8–10 cells). * indicate significantly different values at P ≤ .001 estimated using student t-test.

During the final stages of cell division, plants form a structure called the cell plate that separates the two daughter cells. In *Arabidopsis*, the growing cell plate accumulates a mixture of membrane embedded proteins. These include KNOLLE (SYP111) that originates from *de novo* synthesis and PEN1 that recycles to the cell plate from the PM.^10^ Interestingly, in *Marchantia* MpSYP12A also accumulates at the growing cell plate.^11^ To study this localization behavior further, we again used RFP-PEN1 as an internal control and quantified the relative accumulation of the four SYP12s at the growing cell plate. Remarkably, while we found that PEN1 accumulates strongly at the growing cell plate, the same relative accumulation was not found for neither SYP122, MpSYP12A nor MpSYP12B (Figure 2; Supplementary Fig. S2).

## Discussion

Combined, we show that PEN1 is a highly mobile member of the SYP12 clade, which contrasts the behavior of the other SYP12s tested. This correlates with PEN1ʹs specific ability to mediate pre-invasive immunity to powdery mildew fungi and is strongly suggestive of a mechanistic link. This specialization of PEN1 could reflect the invention of a new syntaxin function in addition to the conserved SYP12 function that mediates a more general immunity.^6^ However, as all the SYP12s accumulate in BFA bodies and at the growing cell plate (albeit not to the same level as PEN1), it is possible that the conserved SYP12 clade function also requires a recycling event. In this scenario, the specialization of PEN1 could reflect an optimization of the existing SYP12 clade function. The specialization of PEN1 is likely based on an alteration of the protein sequence; however, it is difficult to predict the important feature based on the alignment of the four SYP12s alone (Supplementary Fig. S3). In many respects, the specialization of PEN1 offers an intriguing possibility to investigate the molecular mechanisms governing syntaxin mobility.

## Supplementary Material

Supplemental Material

## Data and materials availability

All materials are available on request.
