# Supplementary material for: Mobility of the syntaxin PEN1 in Arabidopsis reflects functional specialization of the conserved SYP12 clade
Source: Plant Signal Behav. 2022 Jun 12;17(1):2084278. doi: 10.1080/15592324.2022.2084278 (PMC9196765; doi:10.1080/15592324.2022.2084278)
Supplement: Supplemental Material [file KPSB_A_2084278_SM3500.pptx]

## Slide 1
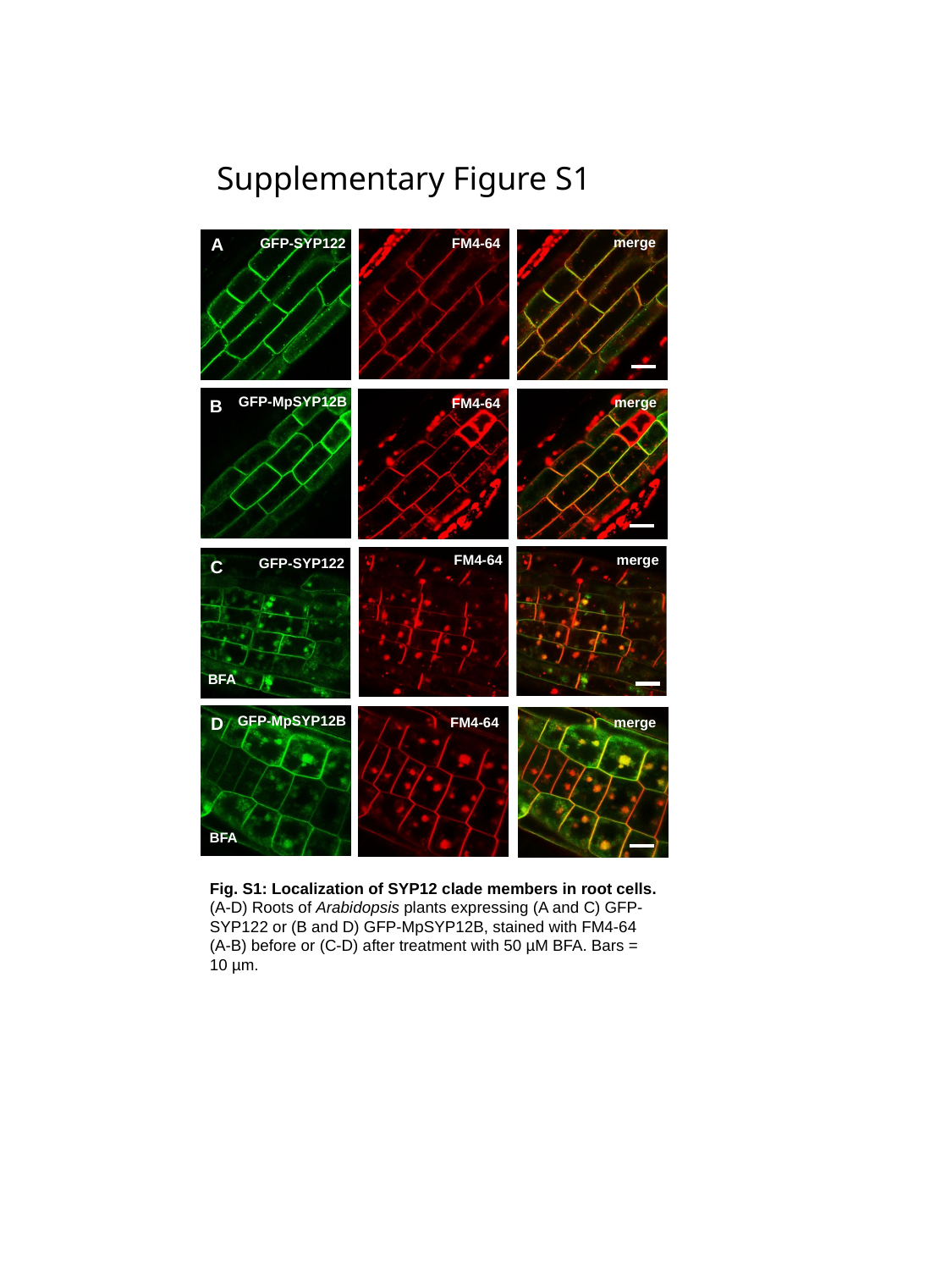

Supplementary Figure S1
A
merge
GFP-SYP122
FM4-64
GFP-MpSYP12B
merge
FM4-64
B
FM4-64
merge
GFP-SYP122
C
BFA
GFP-MpSYP12B
D
FM4-64
merge
BFA
Fig. S1: Localization of SYP12 clade members in root cells.
(A-D) Roots of Arabidopsis plants expressing (A and C) GFP-SYP122 or (B and D) GFP-MpSYP12B, stained with FM4-64 (A-B) before or (C-D) after treatment with 50 µM BFA. Bars = 10 µm.

## Slide 2
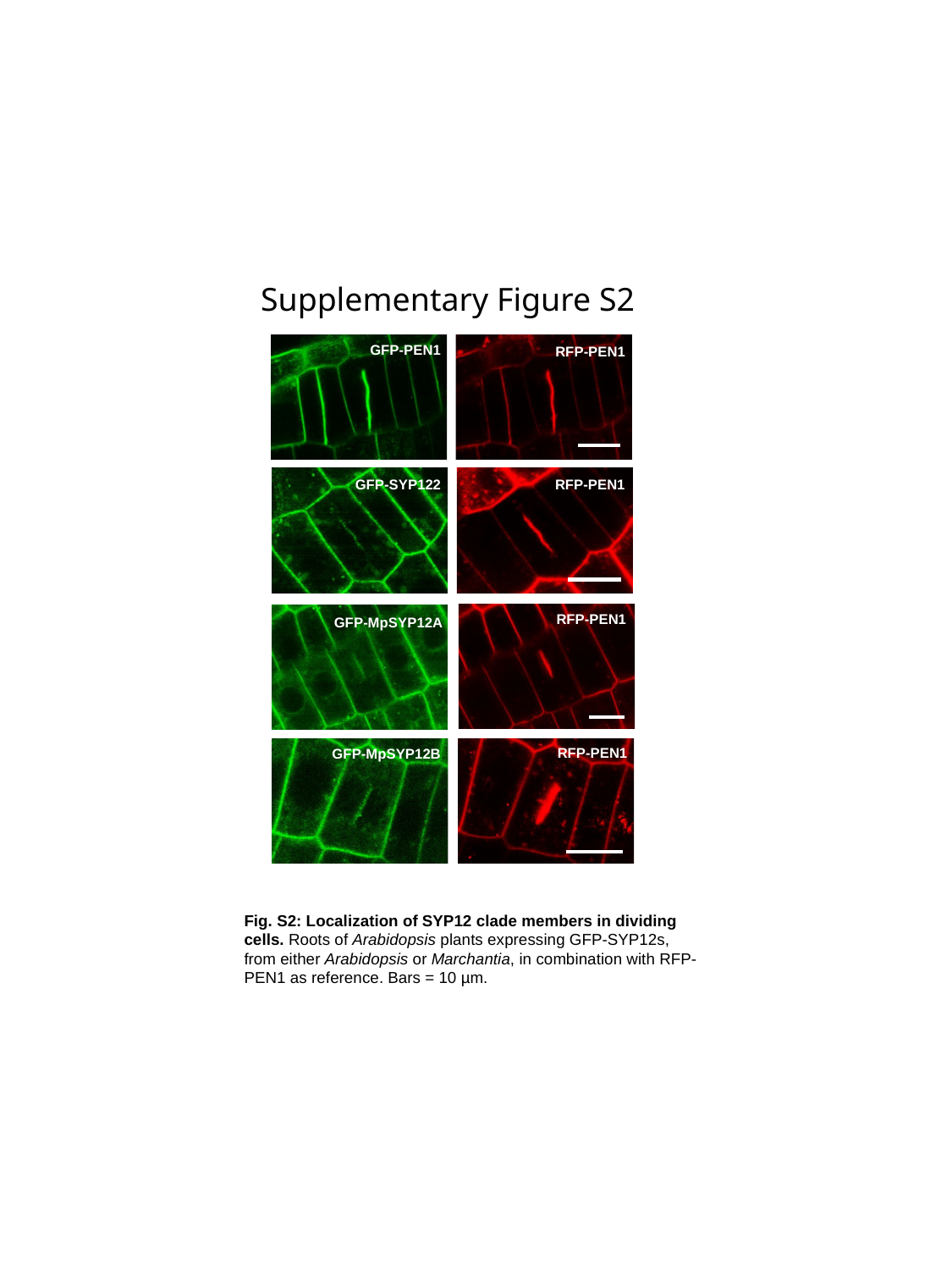

Supplementary Figure S2
GFP-PEN1
RFP-PEN1
GFP-SYP122
RFP-PEN1
RFP-PEN1
GFP-MpSYP12A
RFP-PEN1
GFP-MpSYP12B
Fig. S2: Localization of SYP12 clade members in dividing cells. Roots of Arabidopsis plants expressing GFP-SYP12s, from either Arabidopsis or Marchantia, in combination with RFP-PEN1 as reference. Bars = 10 µm.

## Slide 3
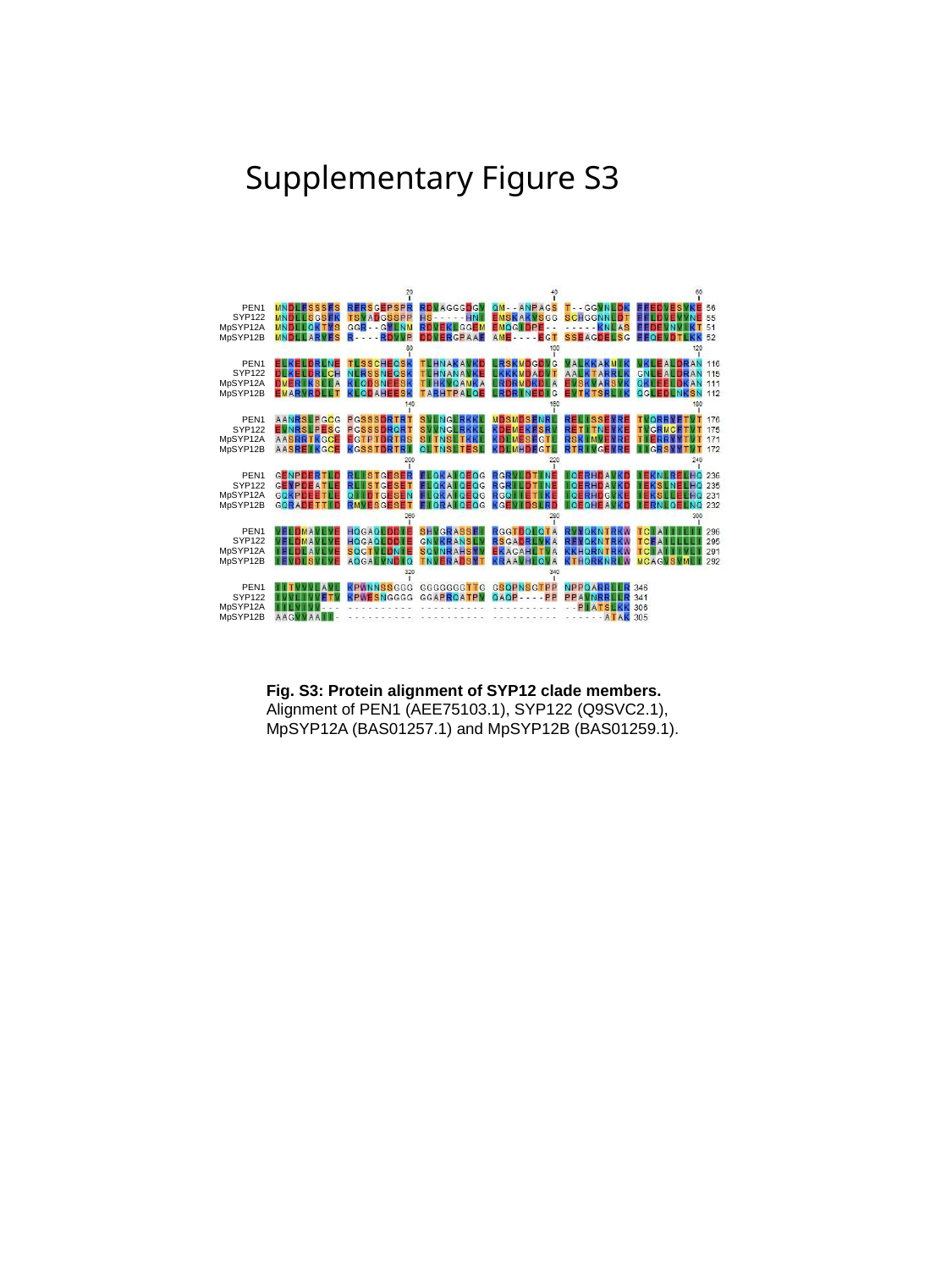

Supplementary Figure S3
PEN1
SYP122
MpSYP12A
MpSYP12B
PEN1
SYP122
MpSYP12A
MpSYP12B
PEN1
SYP122
MpSYP12A
MpSYP12B
PEN1
SYP122
MpSYP12A
MpSYP12B
PEN1
SYP122
MpSYP12A
MpSYP12B
PEN1
SYP122
MpSYP12A
MpSYP12B
Fig. S3: Protein alignment of SYP12 clade members. Alignment of PEN1 (AEE75103.1), SYP122 (Q9SVC2.1), MpSYP12A (BAS01257.1) and MpSYP12B (BAS01259.1).
